# Pediculosis Is a Risk Factor for Iron Deficiency Anaemia

**DOI:** 10.7759/cureus.22403

**Published:** 2022-02-20

**Authors:** Hussain A Al Ghadeer, Fadi Busaleh, Hassan M Albahrani, Alla M Albisher, Abdulaziz AlHassan, Hassan M AlAmer, Hassan M Alibrahim, Ahmed H Alherz, Abdullah F Al Muaibid, Tawfiq M Aljubran

**Affiliations:** 1 Pediatrics, Maternity and Children Hospital, Al-Ahsa, SAU

**Keywords:** saudi arabia, alahsa, profound anaemia., iron deficiency anaemia, head lice, pediculosis capitis

## Abstract

Pediculosis capitis is obligate ectoparasite that lives and feeds on host blood, affecting commonly children. Lice are transmitted easily and respond to topical treatment with good personal hygiene. Chronic infestation can lead to different complications such as bacterial infection dermatitis and anaemia. Haematological complications are not reported frequently. We report a case series of five patients presented with a clear manifestation of anaemia with heavy lice infestation. Laboratory evaluation revealed microcytic hypochromic anaemia (red blood cell indices) with low serum iron levels and other causes that were excluded. All patients who were admitted received blood transfusions. There was not a possible explanation for this severe anaemia other than head lice.

## Introduction

Pediculosis capitis, a common skin condition known as head lice, is considered a general public issue caused by Pediculus humanus (Pediculus humanus capitis) infestation. It is contagious and transmitted through direct contact (head-to-head) or indirect contact through sharing clothes, towels and other personal items [1]. Although it is affecting all age groups but is frequently seen in the first decade of life [2]. The prevalence of pediculosis depends on several factors: socioeconomic, demographic, educational, and hygiene [3]. Pediculus humanus is a holoparasite blood-feeding human ectoparasite that survives on its host [4]. This results in a blood loss of 0.7 mL/day. Although a small amount of blood loss is insignificant in a healthy child, chronic and heavy infestation causes significant blood loss that leads to anaemia [5].

No definitive relationship between iron deficiency anaemia and pediculosis has been found. Limited literatures reported iron deficiency anaemia in patients with heavy and chronic infestation without apparent cause of anaemia. This is the first case series in the paediatric age group that describes five siblings presented with profound anaemia due to lice infestation.

## Case presentation

This case series describes five female siblings aged between 6-13 years presented on the same day to a pediatric emergency department in Maternity and Children's Hospital, Al-Ahsa, Saudi Arabia. They live in an area that is 300 km away from Al-Ahsa with poor socioeconomic status. All of them did not know to have any significant medical or surgical illnesses and there was no family history of inherited, genetic or haematological diseases.

They had a similar clinical presentation with a history of generalized fatigability and pallor for one month (Table 1). It was associated with non-productive cough and pagophagia for four days and one of them had a syncopal attack lasting for a few minutes. It was also associated with head lice infestation for two weeks and subjective fever that responded to paracetamol. They denied any history of dyspnoea, syncope, dizziness, headache, change in bowel habit (diarrhea, constipation, bleeding) or urine (dysuria, frequency, urgency, proteinuria and hematuria). There was no history of ingestion of herbal or chemical medications. They had no history of weight loss, anorexia or eating disorder. They were not known to have an allergy neither they were on regular medication.

**Table 1:** The clinical presentation of the patients

	Patient A	Patient B	Patient C	Patient D	Patient E
Age (years)	6	8	12	12	13
Clinical features					
Symptoms					
Pallor	+	+	+	+	+
Fatigability	+	+	+	+	+
Dyspnoea	+	+	+	+	+
Syncope	-	+	-	-	-
Bleeding	-	-	-	-	-
Menstrual cycle	-	-	-	-	+++
Headache	-	-	+	-	+
Dizziness	-	-	+	-	+
Pagophagia	+	+	+	+	+
Palpitation	+	+	+	+	+
Chest pain	-	-	-	-	-
Weight loss	-	-	-	-	-
Sings					
Pallor	+	+	+	+	+
Jaundice	+	+	+	+	+
Tachypnoea	+	+	+	+	+
Tachycardia	+	+	+	+	+
Hypotension	-	-	-	-	-
Active bleeding (from the scalp)	+	+	+	+	+
Change in urine colour	-	-	-	-	-
Hematemesis	-	-	-	-	-
Organomegaly	-	-	-	-	-

On examination, they looked ill, pale with dark spots distributed all over the body (blood on crust). Notable head lice infestation with copious nits on the scalp, which was associated with scratch marks with injured scalp, was identified. Their vital signs revealed tachycardia and tachypnoea, others were within the normal level. Systemic examination was unremarkable. There was no lice infestation on the rest of the body and pubis other than the head.

Laboratory tests revealed that all of them had a similar finding of microcytic hypochromic anaemia with mild anisocytosis (Table 2). The iron profile showed a low iron level which confirmed the diagnosis of iron deficiency anaemia. The stool culture test for occult blood and ova was negative. The biochemical and coagulation profile was normal. The test was negative for Heinz body, hemoglobin (Hb) H disease.

**Table 2:** Laboratory investigations

Laboratory tests	Patient A	Patient B	Patient C	Patient D	Patient E	
Complete Blood Count (CBC)						Reference level
Haemoglobin	3	3.8	5.6	4.2	2.9	11.5-13.5 g/dL
White blood cells	5.22	5.48	10.12	2.41	11.71	4.5-13.5 x 10^3^/mL
Red blood cells	2.04	2.40	3.63	2.79	2.37	4-5.2 x 10^6^/uL
Haematocrit	11.6	14.3	20.7	15.7	12.5	35-40%
Platelets	389	346	475	292	501	150-350 x 10^3^/mL
Mean corpuscular volume (MCV)	56.9	59.6	57	56.3	52.7	77-86 fL
Mean corpuscular haemoglobin (MCH)	14.7	15.8	15.4	15.1	12.2	27-31 pg
Mean corpuscular haemoglobin concentration (MCHC)	25.9	26.6	27.1	26.8	23.2	31-34 g/dL
Red Cell Distribution Width (RDW)	20.1	18.2	19.2	21	26.9	11.5-15%
Reticulocyte	0.51	1.18	1.05	0.6	2.2	0.5-1
Iron Profile						
Iron	1.9	1.6	2.2	2.01	2.1	Ferritin: 14-79 ng/mL, Iron: 2.8-22.9 mcmol/L
Total iron-binding capacity	64.85	61.22	79.28	73.40	69	45-72 mcmol/L
Blood Chemistry tests						
Blood Urea Nitrogen	5	3.88	3	2.91	3.2	2.6-6.8 mmol/L
Creatinine	42.02	54.30	36.22	54.74	39	<12 years: 27.40-53.93 mcmol/L, <15 years: 39.78-71.61 mcmol/L
Aspartate Aminotransferase (AST)	37	19	19	17	22	18-36 U/L
Alanine Aminotransferase (ALT)	20	21	22	20	11.4	9-25 U/L
Calcium (Ca)	1.95	1.98	2.26	2.18	2.21	2.3-2.6 mmol/L
Sodium (Na)	136	134	140	135	142	136-143 mmol/L
Potassium (K)	3.3	3.4	3.7	3.9	4.05	3.5-5.1 mmol/L
Chloride (Cl)	99	98	103	97	109.8	101-107 mmol/L
Magnesium (Mg)	0.93	0.90	0.74	0.86	0.80	0.86-1.17 mmol/L
Phosphate (Ph)	1.1	1.2	1	1.1	0.96	1.3-1.9 mmol/L
Lactate Dehydrogenase (LDI)	311	251	257	253	277	<10 years: 192-321 U/L, <15 years: 157-272 U/L
Inflammatory Marker						
Erythrocyte Sedimentation Rate (ESR)	30	47	19	27	47	0-10 mm/hr
C-Reactive Protein (CRP)	Negative	Negative	Negative	Negative	Negative	0.1-1 mg/L
Coagulation Profile						
Prothrombin Time (PT)	11.8	12..3	11.8	13	14.3	12.7-16.1 seconds
Partial Thromboplastin Time (PTT)	28.9	31.5	25	35	40	33.9-46.1 seconds
International Normalized Ratio (INR)	0.98	1.1	1	1.03	1.2	0.97-1.30
Haematological workup						
Heinz body	Negative	Negative	Negative	Negative	Negative	
Haemoglobin H	Negative	Negative	Negative	Negative	Negative	
Haemoglobin electrophoresis	Normal	Normal	Normal	Normal	Normal	
Stool analysis- profile						
All normal, no abnormality detected						

They were initially admitted in the Paediatric Intensive Care Unit (PICU), received packed red blood cells (PRBCs) for normalization of haemoglobin levels. Then they were transferred to the general ward, and started on oral ferrous sulfate and folic acid for iron deficiency anaemia and showered with permethrin shampoo for head lice infestation. The patients were discharged on daily ferrous sulfate and permethrin shampoo with regular follow-up with Paediatric Infectious Diseases.

## Discussion

Lice are obligate human ectoparasites that survive and complete their life cycle on the host’s blood and favour humid areas [4]. There are two distinct types of lice species that need humans as a host. Pthirus pubis (pubic lice, carb lice), considered as a sexually transmitted disease (STD), predominantly infests genital hair. Pediculus humanus (P. humanus) is composed of two ecotypes: P. humanus corporis lice infest the body and P. humanus capitis lice infest the head [6,7]. Lice is a health issue worldwide due to its high prevalence and spread through direct or indirect contact. Female lice live up to 30 days on a host, deposit 10 nits daily and survive up to four days on objects that affirm their ease of transmission [1]. They usually affect the age group of 3-11 years particularly in females due to the culture of long hair [2]. In this current series, all of the patients were infested by pediculosis capitis, which are predominantly seen on heads, without apparent lice on other areas like the pubis or body. The patients are female siblings living within the same house and all of them had severe head lice except their parents. Lice are transmitted rapidly among children in the first decade of life through direct contact during playing, sleeping or through indirect contact during sharing personal items.

The clinical presentation of head lice is pruritis which leads to injured scalp, excoriation and lymphadenopathy. Complications of head lice resulting from parasitism are secondary bacterial infection, dermatitis, local post-therapeutic dermatitis and chronic anaemia. Diagnosis is established by the evidence of living lice, nits on hair shafts [8,9]. Dermoscopy is used to differentiate nits comprising nymphs from empty nits (pseudonymphs) [10]. All of our patients presented with symptomatic anaemia in the form of pallor, generalized fatigability, dizziness and pagophagia. Syncopal attack was seen in the elder sister (13 years old) during the period of menstruation, which exacerbated the symptom. As there is a concurrent head lice infestation, with the exclusion of other causes of anaemia, head lice are the causative reason of iron deficiency anaemia through chronic active blood loss by lice.

Management of head lice necessitates the elimination of living lice and nits. Non-pharmacological treatment consists of mechanical removal and combing with a nit comb every 2-3 days to prevent reinfestation. Ensure good personal hygiene, disinfection of clothes at a temperature greater than 53.5°C (128.3°F) and other personal items shouldn’t be shared. Pharmacological treatment consisting of applying topical pediculicide includes pyrethrins permethrin 1% lotion [11]. Although shaving of the scalp is a simple and effective method, it is not recommended because of psychological distress on the child [12]. At the time of presentation, all of our patients received packed red blood cells due to severe symptomatic anaemia and a significant drop in haemoglobin levels. Topical permethrin is applied and it is advised to repeat it after 10 days with general measurements to prevent reinfestation.

## Conclusions

Iron deficiency anaemia is the most common type of anaemia worldwide. Usually, it is caused by dietary insufficiency, malabsorptive diseases like celiac disease or chronic blood loss as in gastrointestinal bleeding. Chronic blood loss is usually related to the gastrointestinal system due to inflammatory conditions or mechanical causes such as a gastric ulcer. Pediculosis capitis is a hematophagic organism that causes minimal blood loss, but a chronic infestation can be associated with a significant blood loss and presented with iron deficiency anaemia as presented in our case. This should alert the physician to correlate the relationship between chronic infestation and hematophagic organisms as a possible cause of iron deficiency anaemia.
